# Protective Effect of Pure Sour Cherry Anthocyanin Extract on Cytokine-Induced Inflammatory Caco-2 Monolayers

**DOI:** 10.3390/nu10070861

**Published:** 2018-07-03

**Authors:** Thi Le Phuong Nguyen, Ferenc Fenyvesi, Judit Remenyik, Judit Rita Homoki, Péter Gogolák, Ildikó Bácskay, Pálma Fehér, Zoltán Ujhelyi, Gábor Vasvári, Miklós Vecsernyés, Judit Váradi

**Affiliations:** 1 Department of Pharmaceutical Technology, Faculty of Pharmacy, University of Debrecen, 4030 Debrecen, Hungary; nguyen.thi.le.phuong@pharm.unideb.hu (T.L.P.N.); fenyvesi.ferenc@pharm.unideb.hu (F.F.); bacskay.ildiko@pharm.unideb.hu (I.B.); feher.palma@pharm.unideb.hu (P.F.); ujhelyi.zoltan@pharm.unideb.hu (Z.U.); vasvari.gabor@pharm.unideb.hu (G.V.); vecsernyes.miklos@pharm.unideb.hu (M.V.); 2 Department of Feed- and Food Biotechnology, Faculty of Agricultural and Food Sciences and Environmental Management, University of Debrecen, 4030 Debrecen, Hungary; remenyik@agr.unideb.hu (J.R.); homoki.judit@agr.unideb.hu (J.R.H.); 3 Department of Immunology, Faculty of Medicine, University of Debrecen, 4030 Debrecen, Hungary; gogy@med.unideb.hu

**Keywords:** anthocyanin, sour cherry, Caco-2, inflammation, permeability, NF-κB

## Abstract

Anthocyanins have several beneficial effects, especially on inflammatory and oxidative conditions. The pro-inflammatory cytokines, tumor necrosis factor-α (TNF-α) and interleukin-1β (IL-1β), induce damage in the intestinal barrier and participate in the pathogenesis of chronic bowel diseases. A number of fruits have high anthocyanin contents with strong biological activity which can support protective actions. Sour cherry (*Prunus cerassus*) is one of the richest fruits in anthocyanins; especially it has high content of cyanidins. The aim of this study was to test the biological effects of a pure sour cherry anthocyanin extract under inflammatory conditions on the intestinal barrier. Caco-2 monolayers were stimulated with 50 ng/mL TNF-α and 25 ng/mL IL-1β, and the protective effects of the anthocyanin extract were examined. We demonstrated the safety of 500, 50, 5 and 0.5 µM anthocyanin extracts through cell impedance measurements. The 50 µM anthocyanin extract inhibited the cytokine-induced Caco-2 permeability and the nuclear translocation of NF-κB p65 subunits. The extract significantly reduced the release of IL-6 and IL-8 production in intestinal cells and glutathione peroxidase activity stimulated by cytokines. We demonstrated, for the first time, the beneficial effects of pure sour cherry anthocyanin extract on inflammatory Caco-2 monolayers, indicating that this substance could be protective in inflammatory bowel diseases and is an excellent raw material for further applications and formulations.

## 1. Introduction

Colorful fruits may be rich sources of anthocyanins, among which the high polyphenol content of the sour cherry is remarkable [1]. Hungarian sour cherry (*Prunus cerassus*) cultivars, such as “*Érdi bőtermő*”, “*Csengődi csokros*” and “*Újfehértői fürtös*” [2,3], contain extremely high levels of anthocyanins as evidenced by studies carried out over the last decade. In our previous study, the anthocyanin compositions of different sour cherry cultivars were determined by matrix assisted laser desorption ionization-time of flight mass spectrometry (MALDI-TOF MS) and the total anthocyanin contents were expressed in mg cyanidin-3-glucoside equivalents. The highest anthocyanin-containing sour cherry cultivar (“*Csengődi csokros*”) was selected and its anthocyanin profile was investigated. As a result of this project, a solid phase extraction procedure was developed for the preparation of pure sour cherry anthocyanin extract, which has the same anthocyanin composition as cherry fruit [2].

Many in vivo and clinical studies deal with the antioxidant, anti-inflammatory, antitumor, and antidiabetic activities and the cardiovascular protective effect of anthocyanins. The topical application of 10% freeze-dried black raspberry gel was investigated and the histologic regression of oral intraepithelial neoplasia and genetically studies certified its beneficial effects [4]. In a randomized phase II trial, strawberry intake (60 g/day for 6 months) significantly decreased the histological grade of precancerous lesions, inhibited cell proliferation and also reduced the protein expression levels of inducible nitric oxide synthase (iNOS), cyclooxygenase-2 (COX-2) and phospho-nuclear factor kappa B (NFκB)-p65 protein expression levels [5]. Twenty colorectal cancer patients were administered 60 g/day of freeze-dried black raspberries orally for 1 to 9 weeks. Treatment with the black raspberry powder protectively modulated both genetic and epigenetic biomarkers in tissues from colorectal cancer patients. This resulted in demethylation of the promoter regions of relevant tumor suppressor genes (such as *SFRP2*, *SFRP5*, and *W1F1*) in adjacent normal tissues and colorectal tumors [6]. The beneficial effects of the oral consumption of freeze-dried black raspberries were investigated in a phase I pilot clinical trial study, where the plasma concentrations of interleukins (IL-1β, IL-2, IL-6, IL-8, IL-10, IL12p70), granulocyte macrophage colony stimulating factor (GM-CSF), interferon-γ, and tumor necrosis factor-α (TNF-α) were measured in 24 colorectal cancer patients. Their results showed an increase in the plasma concentration of GM-CSF and a decrease in IL-8 in patients with the oral consumption of berry product for more than 10 days [7].

The excellent nutritional value and bioavailability of sour cherry cultivars has been investigated by several research groups [3,8]. Earlier studies verified that its low bioavailability leads to quick excretion. A recent study examined the bioavailability of ^13^C labelled cyanidine-3-glucoside in healthy male participants and determined a minimum 12.3 ± 1.3% relative bioavailability. The maximum rate of ^13^C elimination was achieved 30 min after ingestion and the metabolites were present in the circulation for ≤48 h [9]. Based on these data, anthocyanins are more bioavailable than previously described which explains the results of epidemiological studies suggesting the highly beneficial effects of anthocyanins. On the other hand, the bioavailability of anthocyanins depends on several factors. The possibility that anthocyanins contribute to well-being can be explained by the direct effect of absorbed parent anthocyanins through their metabolites and indirect effects that are mediated by non-absorbed anthocyanin derivatives and modified by the microbiome [10]. The low pH range in gastric fluid (pH 1–2) stabilizes anthocyanins (flavylium cation) as confirmed by in vitro digestion studies [11]. In the small intestine, at pH 7.5–8.0, anthocyanins are in the chalcone pseudobase molecular form or bound to pancreatic/bile salts and form insoluble complexes, thus decreasing the bioavailability. However, to achieve anti-inflammatory effect in the gastrointestinal tract (GIT), a sufficiently high local concentration of anthocyanin should be ensured.

The Caco-2 cell line is a human intestinal epithelial cell model that is used for the investigation of intestinal absorption processes and to model intestinal inflammation [12,13,14,15]. The bioactivity of sour cherry anthocyanins was investigated in several studies; in particular, the anti-inflammatory and antioxidant effects were examined [3,16,17]. It is known that the antioxidant and antiproliferative effects and the barrier protection capacity of anthocyanins show chemical structure and conformation dependence [18,19,20]. The structure–activity relationship was confirmed by Cremonini et al. [21]. They compared the membrane barrier protective actions of individual anthocyanin-3-*O*-glucosides and plant extracts that are rich in different anthocyanins. Based on their observations, a significant correlation was established between the transepithelial electric resistance (TEER) and the individual anthocyanins, and the same correlation was detected between the Caco-2 monolayer permeability and individual anthocyanins. The most effective barrier protective extracts were those that contained the highest cyanidin and delphinidin contents. The research group did not find a significant correlation between the TEER and the total anthocyanin content.

In most studies, the bioactivities of individual anthocyanin standards, fruit juice, or fruit flavonoid extracts have been tested; however, the anti-inflammatory effects of purified, native anthocyanins have not been investigated yet. The present work evaluated the anti-inflammatory and antioxidant activity and the protective effects of native pure sour cherry anthocyanin extract on the inflammatory Caco-2 monolayer.

The aims of our study were to investigate the anti-inflammatory and antioxidant activity and the protective effects on the inflammatory Caco-2 monolayer to determine the capacity of native sour cherry anthocyanins. It is important to note that, in fruit juices and smoothies, other components (vitamin C, flavonoids, and organic acid content) modify the bioactivity of the anthocyanins. High anthocyanin-containing foods and nutritional products (food supplements, special formulas, novel foods) formulations have been developed to a large extent in recent years. In this study, the examined native pure anthocyanin extract contained anthocyanins in the original ratio, did not contain other components, and might be a good and safe source of anthocyanins for these nutritional products. Thus, we investigated the new anthocyanin extract to give data for further applications on whether during the extraction procedure preserves the health supportive activities of anthocyanins.

## 2. Materials and Methods

### 2.1. Materials

Pure sour cherry anthocyanin extract (AC) was prepared by the Department of Feed and Food Biotechnology, University of Debrecen, (Hungary), as described earlier [2]. All other reagents were from Sigma-Aldrich (Budapest, Hungary).

### 2.2. Cell Culture

Human Caco-2 intestinal epithelial cells [European Collection of Cell Cultures (ECACC, UK)] were grown routinely in Dulbecco’s Minimum Essential Medium (DMEM), supplemented with 10% fetal bovine serum. Cell monolayers (10–40 passages) were grown on 1.12 cm^2^ permeable Transwell^®^ polycarbonate filters with 0.4 µm pore size (Corning, Lowell, MA, USA), and then used for experimentation following a 21-day culture period to form fully differentiated, confluent monolayers, and polarized into apical (upper) and basal (lower) aspects. These samples were used for the examination of the TEER and the IL-6 and IL-8 concentrations.

**Figure 1:**
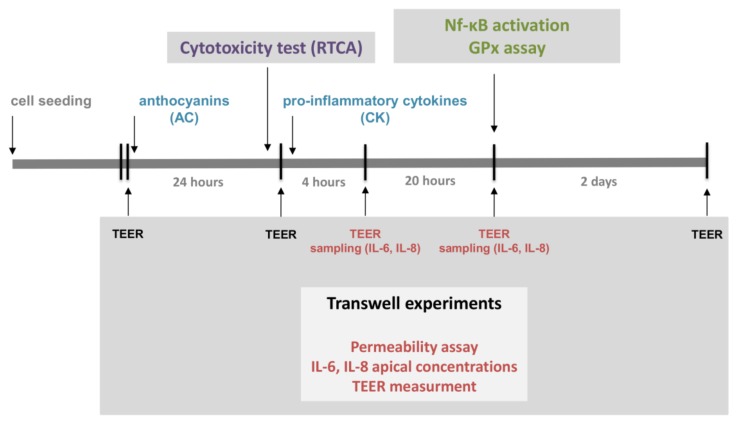
Experimental design of the present study. The investigation began with a cytotoxicity assay of the pure sour cherry anthocyanin extract (AC) with a real-time cell analyser (RTCA). AC pre-treatment and pro-inflammatory stimuli were applied for the investigation of NF-κB activation and glutathione peroxidase (GPx) activity; this was performed in 12-well plates. A permeability assay, Transepithelial Electric Resistance (TEER) measurements and sampling for interleukin-8 (IL-8) and interleukin-6 (IL-6) concentration determination were carried out on transwell plates after AC pre-treatment and pro-inflammatory stimuli.

The experimental design is presented in Figure 1.

### 2.3. Real-Time Monitoring of Cell Index

A real-time cell analyser (RTCA) XCelligence system (Biotech, Hungary) was used to monitor the biological status of Caco-2 cells during anthocyanin treatment. Cellular adhesion was investigated on E-plates, which are coated by gold sensor arrays to measure electrical impedance. The results were converted to the cell index by the XCelligence software (version 1.2.2). Culture medium (100 µL) was added to each well to read the backgrounds, and then 100 µL cell suspensions were dispensed into the wells at a density of 2 × 10^4^ cells/well. Caco-2 cells were kept in an incubator at 37 °C for 7 days. After the cell index reached the maximum values in each well, the cells were treated with different concentrations of anthocyanin extract (AC) and with Triton-X as a positive control [22]. The cell index (CI) was further measured for 48 h.

### 2.4. Measurement of Transepithelial Electric Resistance (TEER)

TEER was employed to investigate the confluency and integrity of Caco-2 cell monolayers which reflects the tight junction functions in inflamed membranes. Caco-2 cells were seeded at a density of 200,000 cells/well on Corning Transwell^®^ polycarbonate filters. Culture medium was replaced with fresh medium every two or three days in the Transwell^®^ inserts. Monolayers were used for the experiments between 20 and 35 days after seeding. To measure the TEER values of the Caco-2 cell layers, Millicell–ERS voltohmmeter electrodes (Merck KGaA, Darmstadt, Germany) were submerged into the apical and basal chambers of the culture medium. In permeability experiments, TEER values were recorded at selected time points, including at the beginning and at the end of sampling to check the monolayer’s integrity and to measure the effects of pre-treatment of anthocyanin extract and cytokines. In these experiments, a 900 Ω cm^2^ TEER value was considered to be a valid indicator for the confluence of a Caco-2 monolayer with sufficient biological membrane barrier integrity to allow it to compartmentalize biological molecules.

Caco-2 cells were cultured without stimulants until the 900 Ω cm^2^ TEER value threshold was reached for each monolayer (indicating biological barrier integrity). Triplicate Caco-2 cell monolayers were treated in the following order: (i) untreated control; (ii) 50 ng/mL TNF-α + 25 ng/mL IL-1β (CK); (iii) 50 µM AC pre-treatment + CK treatment; (iv) 5 µM AC pre-treatment + CK; (v) 0.5 µM AC pre-treatment + CK; (vi) 50 µM AC; (vii) 5 µM AC; and (viii) 0.5 µM AC. TEER values were measured both after the anthocyanin pre-treatment period and 4 h and 24 h after the cytokine treatment to determine stimulating effects on Caco-2 monolayer barrier function.

### 2.5. Measurement of Permeability

Confluent and differentiated cell layers were pre-treated in the upper compartment with 50 µM anthocyanin extract dissolved in cell culture medium for 24 h. Fifty nanograms/milliliter TNF-α + 25 ng/mL IL-1β (CK) were added into the medium and the plates were incubated at 37 °C in an incubator with 5% CO_2_ for 20 h. Caco-2 monolayers were washed and pre-incubated with Hanks’ Balanced Salt solution (HBSS) for 20 min at 37 °C and then incubated with 50 µg/mL Lucifer Yellow (LY) dissolved in HBSS in the upper compartment. Afterwards, the incubation samples were collected from lower compartment at 60, 90 and 120 min, and the volume was supplemented with HBSS. The concentration of the marker molecule was determined with a fluorescence multiwell plate reader (Fluostar Optima, BMG Labtechnologies, Ortenberg, Germany) at 450 nm excitation and 520 nm emission wavelength. The apparent permeability coefficients (P_app_) were calculated with the following equation:

P_app_ = *dQ*/*dt* × 1/(*C*_0_ × *A*) (1)

where P_app_ is the apparent permeability coefficient (cm/s); *dQ*/*dt* is the permeability rate of substances (mol/s); *C*_0_ is the initial concentration of the substances in the upper compartment (mol/mL); and *A* is the surface area of the membrane (cm^2^).

### 2.6. Immunohistochemical Staining and Analysis of Nuclear Factor Kappa Beta (NF-κB) Nuclear Translocation

Caco-2 cells were seeded onto sterile microscope slides at a density of 50,000 cells per slide. Following 6 days of culture in DMEM, the medium was renewed, and cells were pre-treated with 50 µM anthocyanin extract for 24 h. For the induction of inflammation, the cells were exposed to proinflammatory stimulants for 30 min, after which each slide was fixed and stained for the nuclear localization of p65, which is an indicator of NF-κB activation.

This investigation was conducted according to the following design: Caco-2 cultures were divided into four groups according to the stimulant(s) used. The first group of slide cultures was untreated and served as negative controls. The second group of cultures was stimulated with 25 ng/mL IL-1β and 50 ng/mL TNF-α (CK); a third group was pre-treated with 50 µM AC after this with CK; and a fourth group was pre-treated with 50 µM AC and not treated with CK. Following treatment with AC and/or CK, the cells were fixed with ice-cold methanol–acetone (50:50%) for 10 min, and non-specific antibody binding sites were blocked with FBS for 15 min. Next, primary labelling of the NF-κB p65 subunit was conducted using 2 µg/mL rabbit anti-human p65, followed by washing with Hanks Balanced Salt (HBSS) solution. After this, secondary staining with Alexa Fluor 488-conjugated goat-anti-rabbit IgG (Life Technologies, Waltham, MA USA), and Hoechst 33342 (Sigma-Aldrich, St. Louis, MO, USA) was applied. Samples were observed with a Zeiss Axio Scope.A1 fluorescent microscope (HBO 100 lamp) (Carl Zeiss Microimaging GmbH, Göttingen, Germany). Images were analyzed with ZEN 2012 v.1.1.0.0 software (Carl Zeiss Microscopy GmbH, Göttingen, Germany), and the ratio of nuclear and perinuclear fluorescence intensity was calculated.

### 2.7. Flow Cytometric Bead Array Cytokine Assay

The quantification of IL-6 and IL-8 was performed with a multiplexed flow cytometric bead array (CBA) Human Inflammatory Cytokines Kit (Becton Dickinson Biosciences (BD), San Jose, CA, USA). The confluent Caco-2 monolayers were formed on Transwell^®^ inserts. Fifty microliters of Caco-2 culture medium was collected from the apical Transwell^®^ compartments at 4 h and 24 h following the stimulation of Caco-2 monolayers with selected stimulants (AC and/or CK, similar to TEER measurements). The CBA kit was used according to the manufacturer’s instructions. Samples collected for analyses were diluted 2 and 5 times in the kit’s assay buffer. Fifty microliters of diluted samples of appropriate cytokine standards were added to 50 µL of fluorescent cytokine capture bead suspension and 50 µL of human inflammatory cytokine-phycoerythrin (PE) detection reagent on multi-well filter microplates. The plates were incubated for 2 h on a microplate shaker at room temperature, followed by washing with a vacuum filtration manifold. One hundred and twenty microliters of assay buffer was added to each well, and the cytokine content of each sample was measured by FACS array cytometry (BD Biosciences, San Jose, CA, USA). The outcome data were analyzed using Flow Cytomix Pro 2.3 software (Bender MedSystems, Vienna, Austria).

### 2.8. Determination of Glutathione Peroxidase (GPx) Activity

The glutathione peroxidase (GPx) activity of Caco-2 cells was determined with a Glutathione Peroxidase Assay Kit (Cayman Chemical, Ann Arbor, MI, USA). Caco-2 cells were seeded and grown in Costar 12-well Cell Culture Cluster (Corning, Lowell, MA, USA). Cell culture wells were divided into three groups: control (C), CK stimulated and AC pretreated + CK induced samples (AC + CK). Caco-2 cells were treated/pre-treated for 24 h and stimulated with proinflammatory cytokines for 24 h. Cells were washed with PBS and collected with rubber policeman and then centrifuged for 10 min, 2000× *g* at 4 °C. Cell pellets were lysated in cold buffer (50 mM Tris-HCl, pH 7.5, 5 mM EDTA, 1 mM dithiothreitol (DTT)) and centrifuged at 10,000× *g* for 15 min at 4 °C. Removed supernatant was stored on ice, and the assay was performed on the same day as the sample preparation.

### 2.9. Statistical Analysis

For statistical analyses, SigmaStat software (version 3.1; SPSS Inc., Chicago, IL, USA) and GraphPad Prism 5.0 software (GraphPad Software Inc., La Jolla, CA, USA) were used. Data are presented as means ± SDs. Comparisons of groups were performed using ANOVA and Tukey’s Multiple Comparison or Bonferroni tests. Differences were considered significant at *p* < 0.05.

## 3. Results

### 3.1. Real-Time Monitoring of Cell Index

**Figure 2:**
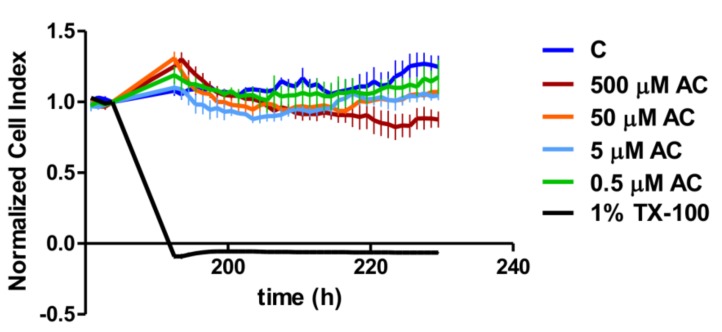
Cell index of Caco-2 cell layers treated with different concentrations of anthocyanin extract (AC): 0.5 µM, 5 µM, 50 µM and 500 µM AC, and with 1% Triton-X-100 (TX-100). Cell suspensions (100 µL) were dispensed into the wells at a density of 2 × 10^4^ cells/well, and then Caco-2 cells were kept in incubator at 37 °C for 7 days. After the cell index had reached its maximum value in each well, the cells were treated, and the cell index was normalized at the time of treatment. Data are presented as means ± SDs, *n* = 4.

The cytotoxicity of the anthocyanin extract (AC) was investigated on Caco-2 cell layers when the cells reached the maximum cell index value (Figure 2). The different concentrations of anthocyanin extract did not show a significant reduction on the cell index, as was discovered for the case of the positive control, 1% Triton-X.

### 3.2. Measurement of Transepithelial Electric Resistance (TEER)

**Figure 3:**
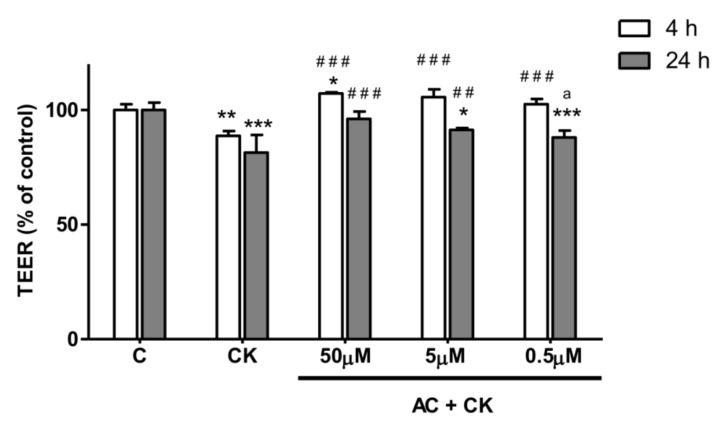
The barrier function of the Caco-2 cell monolayer was investigated with transepithelial electric resistance (TEER). The monolayers were treated with 25 ng/mL IL-1β and 50 ng/mL TNF-α (CK) or pre-treated with 0.5 µM, 5 µM or 50 µM AC for 24 h and then stimulated with cytokines (AC + CK). At 4 h and 24 h after CK induction, the TEER was measured. TEER values are shown as percentages of the untreated control. Results are presented as means ± SDs, *n* = 3; *** *p* < 0.001, ** *p* < 0.01, * *p* < 0.05, ### *p* < 0.001, ## *p* < 0.01. * represents CK and AC + CK compared to C; AC + CK compared to CK—represents #; a represents 0.5 µM AC + CK compared to 50 µM AC + CK.

Cells were grown in medium until a TEER value of 900 Ω cm^2^ was obtained (*n* = 36). Treatment with a combination of TNF-α and IL-1β cytokines decreased the resistance of Caco-2 monolayers to 88.80% (*p* < 0.01) after 4 h and 81.50% (*p* < 0.001) 24 h later (Figure 3) compared to the TEER changes in the control and untreated group. Twenty-four hours of pre-treatment with different concentrations of anthocyanin extract caused a significant difference (*p* < 0.001) in TEER values after 4 h of cytokine treatment (AC + CK) compared to the CK group. However, 24 h later, the TEER values showed an anthocyanin dose dependent response to cytokine treatment: 50, 5 and 0.5 µM AC pre-treatment led to values that were 96.1%, 91.32% and 88.05% TEER of the control, respectively.

### 3.3. Measurement of Permeability

**Figure 4:**
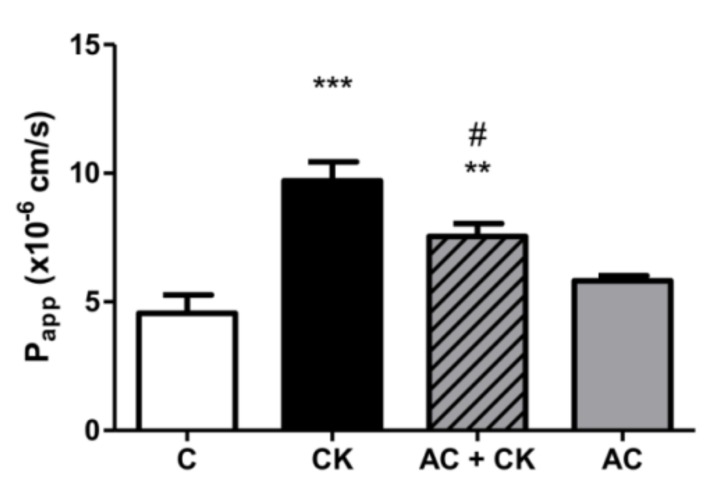
The barrier function of the Caco-2 cell monolayer was investigated as the permeability of Lucifer Yellow (LY), and the apparent permeability coefficient (P_app_) was calculated. Caco-2 monolayers grown on culture inserts were treated with 25 ng/mL IL-1β and 50 ng/mL TNF-α (CK) or pre-treated with 50 µM AC for 24 h; then permeability was measured for LY. The control group (C) received the culture medium. Results are presented as means ± SDs, *n* = 6. *** *p* < 0.001, ** *p* < 0.01, # *p* < 0.05. CK and AC + CK compared to C—represents *; AC + CK compared to CK—represents #.

The permeability of Lucifer Yellow, a small paracellular marker, was measured from the apical to the basal side (Figure 4). The Caco-2 cell layer permeability significantly increased after CK induction from 4.56 × 10^−6^ to 9.71 × 10^−6^ cm/s (*p* < 0.001). AC pre-treatment caused a significant reduction (from 9.71 × 10^−6^ to 7.54 × 10^−6^ cm/s; *p* < 0.05) in cell layer permeability compared to treatment with CK.

No significant differences were noted between the single AC treatment and the untreated Caco-2 cell layer permeability (5.82 × 10^−6^ cm/s).

### 3.4. Flow Cytometric Bead Array Cytokine Assay

**Figure 5:**
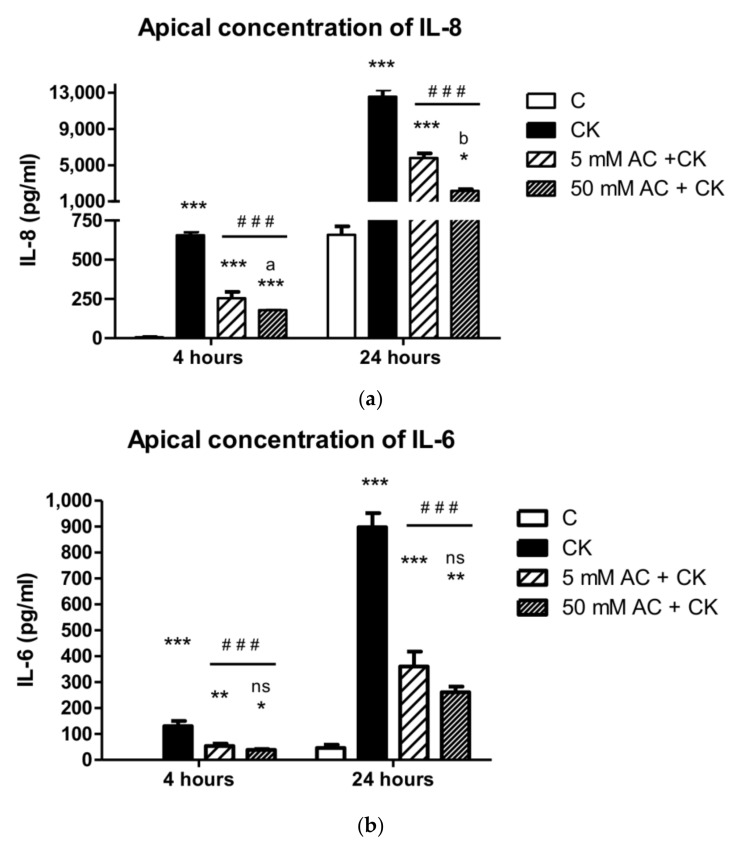
Apical concentration of IL-8 (**a**) and IL-6 (**b**) after CK induction in medium. The control group (C) received culture medium. The monolayers were treated with 25 ng/mL IL-1β and 50 ng/mL TNF-α (CK) and pre-treated with 5 µM or 50 µM AC. Results are presented as means ± SDs, *n* = 3–5, *** *p* < 0.001, ** *p* < 0.01, * *p* < 0.05, ### *p* < 0.001. * represents CK and AC + CK compared to C; AC + CK compared to CK—represents #; a represents 5 µM AC + CK vs. 50 µM AC + CK, *p* < 0.05; b represents 5 µM AC + CK vs. 50 µM AC + CK, *p* < 0.001.

Cytokine induction of the Caco-2 cell layer caused significant IL-8 and IL-6 release (*p* < 0.001) into apical compartments after both the 4 h and 24 h induction times (Figure 5). In comparison to CK stimulated cultures, 24 h of AC pre-treatment resulted in significantly decreased IL-8 and IL-6 release (*p* < 0.001) after 4 h and 24 h CK induction. However, IL-8 and IL-6 release did not decrease to control levels, and there were significant differences (*p* < 0.001) after both the 4 h and 24 h induction times. Fifty micromole AC and 5 µM AC pre-treatments showed significant concentration dependence on IL-8 levels after 4 h (*p* < 0.05) and 24 h (*p* < 0.01) of CK induction. No statistical differences were observed in IL-6 release at either time point (*p* > 0.05).

### 3.5. Immunohistochemical Staining and Analysis of Nuclear Factor Kappa Beta (NF-κB) Nuclear Translocation

**Figure 6:**
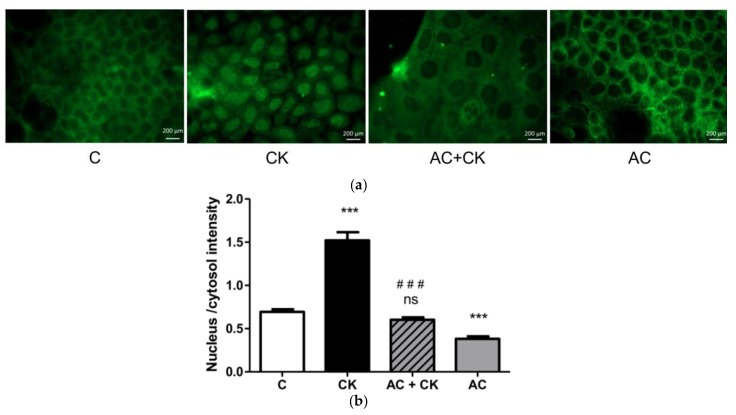
Immunohistochemical staining (**a**) and analysis (**b**) of NF-κB activation. Caco-2 cells were treated for 30 min with culture medium (C), pro-inflammatory cytokines (CK; 50 ng/mL TNF-α and 25 ng/mL IL-1β), and/or pre-treated with 50 µM AC. Nuclear localization of the NF-κB p65 subunit was monitored by immunostaining. Green: p65 staining. Nucleus/cytosol intensity mean: ratio of the fluorescence intensity of the NF-κB immunostaining in the cell nuclei and cytoplasm. Data are presented as means ± SDs, *n* = 8–10. *** *p* < 0.001, ### *p* < 0.001. CK and AC + CK compared to C—represents *; AC + CK compared to CK—represents #.

The activation of the NF-κB pathway through translocation of the p65 subunit of NF-κB indicates the intensity of an inflammatory reaction at the cellular level. The short term (30 min) cytokine treatment of cells induced the translocation of the green-stained p65 subunit from the cytosol to the nuclei which resulted in a high nucleus/cytosol intensity ratio compared to the control group (*p* < 0.001) (Figure 6). A receded appearance of green-stained p65 in the nuclei and an increased intensity of dye in the cytosol were observed in the case of pre-treatment with 50 µM AC corresponding to the CK treated group. The nucleus/cytosol intensity ratio of AC + CK decreased to the control (C) level, and there was no significant difference between them (*p* > 0.05), while the high significance of the nucleus/cytosol intensity between the AC + CK and CK groups predicts the effective inhibition of the cytokine-induced NF-κB pathway (*p* < 0.001). Interestingly, the AC treatment without CK induction caused decreased green-staining in the nuclei; thus, the nucleus/cytosol intensity ratio compared to the control group was significantly reduced (*p* < 0.001).

### 3.6. Determination of GPx Activity

**Figure 7:**
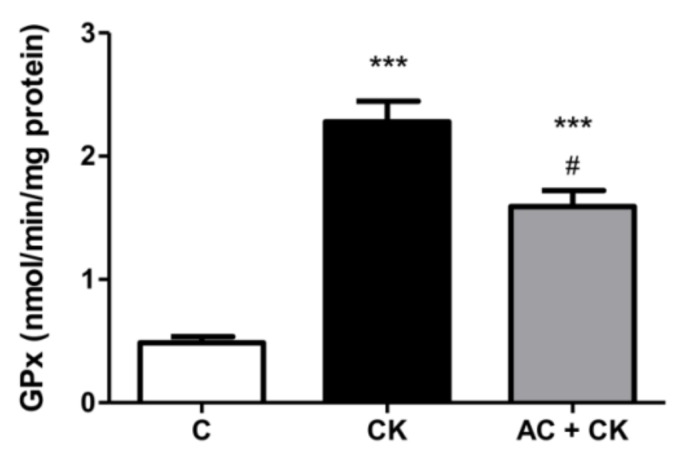
GPx enzyme activity in Caco-2 cells. Caco-2 cells were treated for 24 h with culture medium (C), pro-inflammatory cytokines (CK; 50 ng/mL TNF-α and 25 ng/mL IL-1β), or pre-treated with 50 µM anthocyanins before CK treatment (AC + CK). AC pre-treatment significantly reduced the cytokine-induced GPx activity in the cells. Data are presented as means ± SDs, *n* = 3. *** *p* < 0.001, # *p* < 0.05. CK and AC + CK compared to C—represents *; AC + CK compared to CK—represents #.

GPx enzyme activity after CK induction significantly elevated by more than 4.7-fold (*p* < 0.001), compared to control cells (from 0.485 to 2.28 nmol/min/mg protein) (Figure 7). However, as a result of AC pre-treatment, after the CK induction, GPx activity increased by just 3.2 times in Caco-2 cells (*p* < 0.05) compared to the control. Pre-treatment with anthocyanins significantly reduced GPx activity on inflamed Caco-2 cell cultures, but the enzyme activity did not decrease to control levels.

## 4. Discussion

This study examined the anti-inflammatory, antioxidant and protective action of pure anthocyanin extract (AC) derived from sour cherry on cytokine-induced barrier dysfunction. The most important anthocyanins were identified earlier in five different Hungarian sour cherry cultivars by MALDI-TOF MS analysis. The total anthocyanin content (TAC) of sour cherry extract was 261 mg/100 g fresh fruit [2]. The anthocyanin composition of the applied pure anthocyanin extract was measured by HPLC and was the following: cyanidin-3-*O*-rutinoside (60%), cyanidin-3-*O*-monoglucoside (35%), cyanidin-3-*O*-glucosylrutinoside (0.5%) (see also Supplementary Materials Figure S1). The anthocyanin content, expressed in cyanidin-3-*O*-glucoside, was calculated for molar concentrations during treatments. In previous studies, cyanidin-3-*O*-glucosides have been used in the 0.25–50 µM concentration range on Caco-2 cell monolayers [21,23,24]. Thus, to investigate the cytotoxicity of pure anthocyanin extract on Caco-2 cell cultures, 500, 50, 5 and 0.5 µM concentrations of AC were applied.

At first, we tested the toxicity of the applied AC concentrations. The cytotoxicity effect of AC was investigated by real-time cell analysis on the plateau section of the proliferation curve after the Caco-2 cell layer had reached the maximum value of the CI. The cell index decreased significantly in 1% Triton-X treated samples compared to the control and AC treated groups. Despite there being no significant concentration dependence in the investigated concentration range, the highest concentration (500 µM) of AC treatment resulted in a significant CI reduction compared to the control curve. However, this reduction was less than that caused by 1% Triton-X, because the Bonferroni’s Multiple Comparison Test showed a significant difference (*p* < 0.001) between them. Thus, according to the change in CI, the AC treatments were not regarded as cytotoxic, and for further analysis, we used 50, 5 and 0.5 µM of AC, as used by other research groups [23].

To test the barrier protection effect of AC, we used the Caco-2 inflammatory model [25]. Caco-2 monolayers were treated on their basal compartment with proinflammatory cytokines in the permeability study. Interestingly, no significant TEER decrease was detected when stimuli were applied in the apical compartment, but in the case of basolateral treatment with 25 ng/mL IL-1β, it was effective [13]. This observation might be explained by the polarized localization of cytokine receptors on Caco-2 cells. In our experiments, 25 ng/mL IL-1β, in combination with 50 ng/mL TNF-α, significantly reduced the TEER (20%) after 24 h of stimulation. Different concentrations of AC pre-treatment caused the dose dependent response in TEER value. Additionally we examined the survival rate of Caco-2 cells by RTCA after removal of pro-inflammatory stimuli. We did not observe a significant difference between the cell indexes of CK treated and AC pre-treated samples, indicating that these treatments had no effect on the survival of cells. The reduced barrier damage, reflected in the TEER due to AC is consistent with the results published in previous studies [21]. Different purified anthocyanins (ACs) and anthocyanin rich plant extract (ACRE) were added to the apical compartment of the Caco-2 cell monolayer before stimulation with TNF-α in the basolateral chamber. It has been described that cyanidins and delphinidins are more active than other pure ACs in the prevention of TNF-α induced TEER decrease and the increase of paracellular transport. These results suggest that the protective effect of purified ACs is strongly determined by their chemical structures and conformations. In the case of ACRE treatment, the TEER results predicted the cyanidin content of ACRE, and indeed, the highest cyanidin containing extract showed higher TEER reduction after TNF-α stimulation.

This protective effect was confirmed in our permeability experiments, but with pure anthocyanins extracted from sour cherry. Lucifer Yellow, a small paracellular marker, was used to test the permeability of the monolayer and was determined in the basolateral compartment. A more than twice-fold permeability increase was found in CK-stimulated samples compared to the controls. These data indicate a correlation with our previous results, though we used different markers [25]. AC pre-treatment resulted in a significant decrease in permeability compared to the CK stimulated samples. Cremonini et al. observed that one of the most protective ACs at the lowest concentration (0.25 µM) is cyanidin glucoside, and this correlates with the ACRE protective effect. Our data are in accordance with previous investigations reporting the protective effects of anthocyanins in membrane permeability studies [21]. Both the TEER and permeability results show that pro-inflammatory stimuli cause the disruption of barrier function, but AC prevents this.

The exposure of Caco-2 cells to TNF-α and IL-1β cytokines induces several signalling pathways, such as the NF-κB pathway which has effects on the membrane permeability and tight junction redistribution [26,27]. Activation of NF-κB pathway is a key event in inflammatory reactions, leading to proinflammatory mediator expression, and results in damage to the intestinal barrier. Proinflammatory cytokines promote the expression of two genes coding for TNF-α and IL-8, while cyanidin-3-*O*-glucoside (C3G) pre-treatment reduces the TNF-α and IL-8 mRNA levels [24]. The TNF-α and IL-8 genes contain binding sites for NF-κB; thus, if the TNF-α and IL-8 levels decrease following the pre-treatment of C3G, the NF-κB pathway is inhibited. This hypothesis may explain the results of recent studies in connection with immunohistochemical staining and analysis of NF-κB nuclear translocation. Figure 6 shows a significantly increased fluorescent intensity ratio in cell nuclei after CK induction compared to the control samples; nevertheless, AC pre-treatment decreased this ratio near to the control ratio. These results confirm the NF-κB activation and p65 translocation to the nuclei in the CK treated samples and the inhibition of activation and translocation in AC pre-treated samples. According to our results, pure AC, which is a cyanidin mixture extracted by a special procedure indeed has an anti-inflammatory effect. Some previous studies have shown [21] that C3G is one of the strongest natural anthocyanin inhibitors of NF-κB. Our experiments show that the isolated pure cyanidin mixture has a similarly potent inhibitory effect on the NF-κB activation pathway.

Proinflammatory cytokines induce the NF-κB activation pathway, and consequently, epithelial cell secrete cytokines which contribute to inflammatory conditions. The decreased TEER of the monolayers and increased IL-8 production have been established, indicating epithelial barrier opening and an inflammatory response [13,28]. We observed vectorial and increased IL-6 and IL-8 levels in apical and basal compartment in our previous study [25], and the cytokine concentrations were greater in the apical compartment as reported by other research groups [14]. Because of this significant difference, we determined IL-6 and IL-8 levels in the apical compartment in the present study. Pre-treatment with 5 and 50 µM AC extracts inhibited IL-8 and IL-6 secretion after 4 h and 24 h of pro-inflammatory cytokine induction. In another study, pre-treatment with grape extract resulted in a greater than 50% decrease in IL-8 production on Caco-2 cells after TNF-activation [29]; however, the inhibitory effect of sour cherry extract has not been investigated yet. According to our results, our data can also be compared with the effects of black carrot, purple root vegetables which have the same cyanidin glucoside content as sour cherries. The published decrease in IL-8 and IL-6 production is 40–60% after anthocyanin extract pre-treatment and cytokine induction, compared to cytokine induced samples [29,30], thus we can conclude that the pure sour cherry AC extract has a more pronounced effect on cytokine production.

The glutathione system (GSH/GSSG) is the main redox buffer and indicates the redox condition of cells [31]. The GSH/GSSG ratio in human cells is approximately 500:1; however, under stress conditions, it shifts because of the increased GPx activity. Glutathione peroxidase catalyzes the reduction of peroxide and thus protects cells from oxidative damage. The radical scavenging activity of polyphenols increases with the number of hydroxyl and methoxyl groups in the B rings of molecules. Additionally, an ortho dihydroxyl structure in flavonoids generates a higher antioxidant capacity [32]. These structural characteristics may account for reduced GPx activities in our AC pre-treated samples compared to CK induced samples. In the examined pure AC containing extract, the main components were cyanidin glucosides. In accordance with our results, a high antioxidant capacity was certified in the different cyanidin-glucoside containing samples [18,33].

The abovementioned protective effects of pure AC extract support its possible nutraceutical application, but the development of an appropriate formulation is needed. Since AC extract could have beneficial effects on irritable bowel syndrome and inflammatory bowel diseases, a protective delayed release formulation to deliver AC into the small intestine and the colon should be formulated.

## 5. Conclusions

In conclusion, we confirmed that the pure sour cherry AC extract has anti-inflammatory and barrier protective effects on cytokine-induced inflammatory Caco-2 monolayers. The pure AC extract was prepared by processing fresh sour cherry and did not contain any other natural components. This pure AC extract could be an excellent raw material for further applications and formulations.

## Supplementary Materials

The following are available online at http://www.mdpi.com/2072-6643/10/7/861/s1, Figure S1: HPLC chromatogram of pure sour cherry anthocyanin extract (AC). Figure S2: Normalized cell index after removal of pro-inflammatory stimuli.
