# Supplementary material for: Protective Effect of Pure Sour Cherry Anthocyanin Extract on Cytokine-Induced Inflammatory Caco-2 Monolayers
Source: Nutrients. 2018 Jul 3;10(7):861. doi: 10.3390/nu10070861 (PMC6073755; doi:10.3390/nu10070861)
Supplement: Supplementary file 1 [file nutrients-10-00861-s001.pdf]

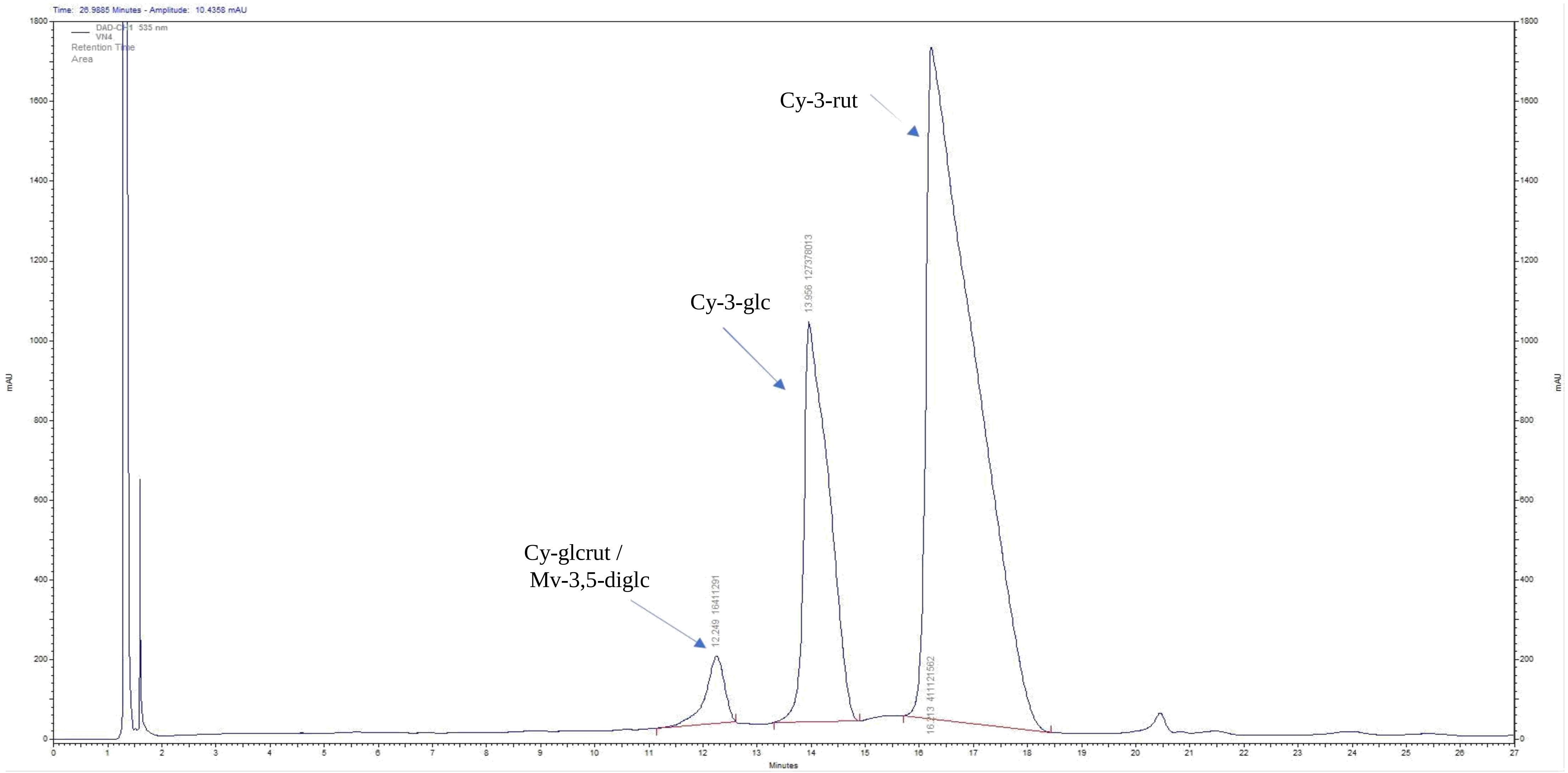

Figure S1. HPLC chromatogram of pure sour cherry anthocyanin extract (AC). Cy-3-rut: Cyanidin-3-O-rutinoside: 60%; Cy-3-glc: Cyanidin-3-O-monoglucoside: 35%; Cy-glcrut/Mv-3,5-diglc: Cyanidin-3-O-glucosylrutinoside/Malvidin-3,5-diglucoside: 0,5%.

- Cy-3-rut: Cyanidin-3-O-rutinoside: 60%
- Cy-3-glc: Cyanidin-3-O-monoglucoside: 35%
- Cy-glcrut/Mv-3,5-diglc: Cyanidin-3-O-glucosylrutinoside/Malvidin-3,5-diglucoside: 0,5%

Cy-3-rut: Cyanidin-3-O-rutinoside
Cy-3-glc: Cyanidin-3-O-monoglucoside
Cy-glcrut/Mv-3,5-diglc: Cyanidin-3-O-glucosylrutinoside/Malvidin-3,5-diglucoside
